# Are we ready to detect nematode diversity by next generation sequencing?

**DOI:** 10.1002/ece3.2998

**Published:** 2017-04-27

**Authors:** Thomas Peham, Florian M. Steiner, Birgit C. Schlick‐Steiner, Wolfgang Arthofer

**Affiliations:** ^1^ Molecular Ecology GroupInstitute of EcologyUniversity of InnsbruckInnsbruckAustria

**Keywords:** DNA extraction kit, environmental DNA, nematode detection, primer fitting

## Abstract

In a Technical Advance article, Porazinska et al. (2009, Molecular Ecology Resources, 9, 1439–1450) assessed next generation sequencing (NGS) as a method for metagenomic analysis of nematode diversity. We agree that NGS has great potential here. However, it is not an easy path to the successful implementation of NGS for environmental DNA analysis of nematodes. Here, we describe the method's limitations and discuss prospective research questions. For instance, only a few direct extraction kits are suitable for nematode DNA extraction from bulk samples without adaptation. They enable the analysis of extracellular nematode DNA. The most crucial and unresolved issue remains the limited availability of suitable primers.

## 1 Nematode Diversity

Nematodes are among the most diverse phyla on earth (Zhang, 2013). Soil pushes this diversity even further with its nested set of ecological worlds (Giller, 1996) and also hides it from us at the same time (Wall, Bardgett, & Kelly, 2010). Compared with conventional, morphology‐based species determination methods, molecular techniques, and metagenomic approaches promise a fast insight into this hidden diversity. They enable us to identify drivers of biodiversity or trophic interactions with advanced speed and resolution. Despite this promising prospect and some encouraging first results, there are several technical and methodological hurdles yet to be overcome. In this comment, we focus on soil DNA extraction kits, sampling of environmental DNA, and the suitability of the primers proposed by Porazinska et al. (2009).

## 2 Successful DNA Extraction as a Starting Point

Today, two different paths for DNA extraction from soil are commonly used. Organisms are either separated from the soil matrix before DNA extraction or used as crude samples including the soil matrix. Traditional nematology developed various techniques for the separation of animals from large‐scale soil samples about 250 ml sized (OEPP/EPPO, 2013). In contrast, kits for metabarcoding assays typically extract total DNA from much smaller samples ranging from 0.25 g (e.g., NucleoSpin^®^ Soil, Macherey‐Nagel, Düren, Germany; Taberlet et al., 2012) to 10 g (e.g., PowerMax^®^ Soil DNA Isolation Kit, MO BIO Laboratories, Carlsbad, CA, USA; Andersen et al., 2012). Each separation and extraction method introduces a bias, as the determined species counts and ratios will differ from those in the soil. Users should be aware of this issue when choosing a method (van Bezooijen, 2006).

**Figure 1:**
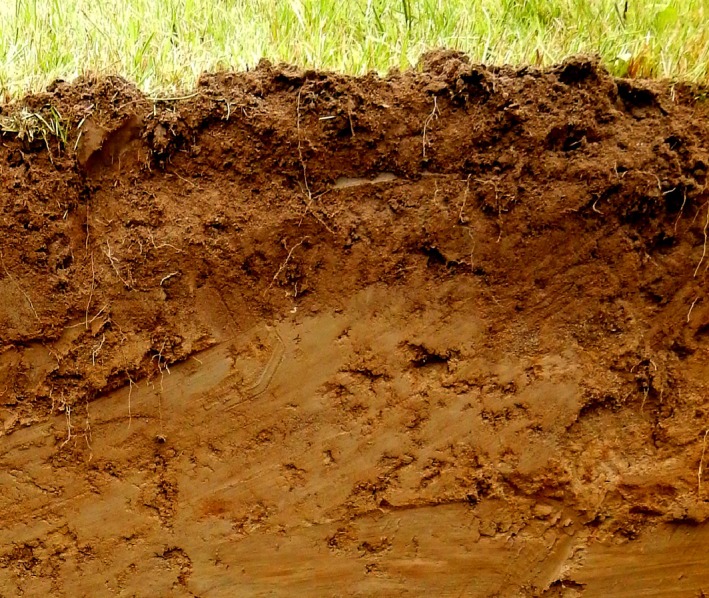
Soil profile of the investigated meadow, a Fluvisol

The first hurdle to be overcome is the low number of soil extraction kits suitable for soil nematodes. As there is a wide range of PCR inhibitors, which highly correlate with the processed matrix (Schrader, Schielke, Ellerbroek, & Johne, 2012), we preselected DNA extraction kits developed specifically for soils. In our experiments, only two of six kits provided extracts containing detectable amounts of nematode DNA (see Box 1, Figure 1). This was surprising, especially because one of the here tested extraction kits (PowerLyzer^®^ DNA Isolation Kit, MO BIO Laboratories) was successfully used in a previous nematode biodiversity study (Sapkota & Nicolaisen, 2015) but failed in our experiments. Reasons for the differing results may be manifold. As extraction control, individuals of *Drosophila nigrosparsa*, an alpine fly not occurring at the sampling sites, was added to the soil before homogenization and DNA extraction. Using highly species‐specific microsatellite primers, the fly DNA was detectable in all extracts. Therefore, insufficient homogenization as well as insufficient removal of PCR inhibitors can be excluded. Differences in the lysis efficiency and in the performance of the washing and elution steps of the various extraction kits were probably a reason for limited extraction success. High purification success, that is, removal of all inhibiting substances, might eventually lead to the removal of substantial amounts of target DNA, too. As the two successful extraction kits required the lowest load of soil of all kits (Table 1), an insufficient sample size can be ruled out as a source of error. Nematodes and drosophilids have a dissimilar cuticle composition (collagen and chitin, respectively); this might make their DNA differently accessible during mechanical and chemical breakup, which can result in different amounts of target DNA. A method to improve the mechanical breakup is described by Sapkota and Nicolaisen (2015) who pretreated their samples by grinding the freeze‐dried soil in a mill for ten minutes instead of following the instructions provided with the kit.

Box 1 **Direct extraction of nematode DNA from soil samples**

1 DNA can be extracted directly from soil samples. Typical extraction kits employ several steps of homogenization, cell lysis, binding of DNA on a membrane, washing, and elution.

We tested six commercially available DNA extraction kits for their suitability to extract nematode DNA directly from soil: NucleoSpin^®^ Soil (Macherey‐Nagel, Düren, Germany), Precellys^®^ Soil DNA Kit (Bertin Technologies, Montigny‐le‐Bretonneux, France), PowerLyzer^®^ Soil DNA Isolation Kit (MO BIO Laboratories, Carlsbad, CA, USA), PowerSoil^®^ Soil DNA Isolation Kit (MO BIO Laboratories, Carlsbad, CA, USA), PowerMax^®^ Soil DNA Isolation Kit (MO BIO Laboratories, Carlsbad, CA, USA), and E.Z.N.A. Mag‐Bind^®^ Soil (Omega bio‐tek, Norcross, GA, USA; see Table 1 for details). They were chosen because they are specific to soil matrices and/or environmental samples, are produced by well‐known companies, and cover the available range of loading capacity (0.25–10 g). All of them were used according to the manufacturer's protocol, and two replicates were made. Ten soil cores of 0–10 cm depth and 2 cm diameter were taken from a meadow (47°15′50,70″N; 11°20′27,85″E; 578 m above sea level; Fluvisol; Figure 1) and mixed thoroughly. 25 g of soil was used for the experiment. To evaluate the successful extraction of DNA, the original soil sample was spiked with 10 individuals of *Drosophila nigrosparsa*. This species was used because it does not occur on that altitude, and because species‐specific microsatellite primers are available (Genomic Resources Development Consortium et al., 2015). For detecting DNA of *D. nigrosparsa*, the primers DN34/F and DN34/R (Genomic Resources Development Consortium et al., 2015) were used, for nematode detection the general nematode primers supplied by Clear^®^Detections (nonpathogenic nematode families: real‐time PCR identification and detection kit; Clear^®^Detections, Wageningen, the Netherlands) were used. The PCR consisted of a denaturation at 95°C for 3 min, followed by 35 cycles of denaturation at 95°C for 10 s, annealing at 63°C for 60 s, and extension at 72°C for 30 s. Amplification success was evaluated by gel electrophoresis. Although the DNA of *D. nigrosparsa* was found in each extract, only the extracts of the Precellys^®^ Soil DNA Kit and Mag‐Bind^®^ Soil DNA Kit delivered nematode PCR products (Table 1).

**Table 1:** Results of nematode DNA extraction with several extraction kits

Soil DNA kit	Company	Max. load (g)	Handling time (min)	*Drosophila nigrosparsa*	Nematodes
NucleoSpin^®^ Soil	Macherey‐Nagel	0.50	30	✓	
Precellys^®^ Soil DNA Kit	Bertin Technologies	0.25	35–55	✓	✓
PowerLyzer^®^ DNA Isolation Kit	MO BIO Laboratories	0.25	30	✓	
PowerSoil^®^ DNA Isolation Kit	MO BIO Laboratories	0.25	30	✓	
PowerMax^®^ Soil DNA Isolation Kit	MO BIO Laboratories	10.00	30	✓	
E.Z.N.A. Mag‐Bind^®^ Soil	Omega bio‐tek	0.25	75	✓	✓

✓ stands for successful PCR amplification.

## 3 The Potential of Environmental DNA

The successful extraction of nematode DNA from bulk soil opens the door to nascent sampling strategies like environmental DNA extracted from samples without obvious biological source material (e.g., water; Thomsen & Willerslev, 2015) or extracellular DNA found in biogenic matter outside living cells (e.g., adsorbed to soil particles; Lorenz & Wackernagel, 1994). Occurrence of extracellular DNA has been proven for soil microorganisms (Smithies & Gibbons, 1955) and plants (Taberlet et al., 2012), but up to now not for nematodes. Here, we are the first to prove that extracellular nematode DNA can be found in bulk extracts of grassland and forest soil (see Box 2). The usual problems with soil heterogeneity (using large sample sizes of up to 2 kg) and seasonality (by representing a long‐time reservoir) are solved by this.

Box 2 **Detection of nematodes via extracellular DNA**

1 Two plots of 100 m² size and about 200 m apart, one on a meadow (47°15′50,70″N; 11°20′27,85″E; 578 m above sea level; Fluvisol) and one in a forest (47°15′45,41″N; 11°20′32,75″E; 579 m above sea level; Fluvisol), were sampled. Two replicates, each consisting of 100 soil cores of 0–10 cm depth and 2 cm in diameter, were taken following a 50‐cm shifted quadratic grid per plot. Phosphate buffer (0.12 mil/L; pH ≈ 8; 1.97 g NaH_2_PO_4_ and 14.7 g Na_2_HPO_4_/L) was added to the soil following the instructions of Taberlet et al. (2012) with a weight ratio of 1:1 (soil:buffer) for the meadow and 1:2 for the forest soil and gently shaken on an Infors HT Multitron shaker (Infors AG, Bottmingen, Switzerland) at 100 rpm for 20 min. A 2‐ml aliquot from the centre of the soil buffer suspension was removed, centrifuged at 10,000 rcf for 10 min, and the supernatant was transferred to a new vial and further processed with the Precellys^®^ Soil DNA Kit, skipping the lysis step. The extracts were finally 1:10 diluted in deionized water. A PCR was performed with general nematode primers from Clear^®^Detections (for PCR settings see Box 1) as well as with 18S and 28S primers from Porazinska et al. (2009) (18S: denaturation at 94°C for 10 min, followed by 35 cycles of denaturation at 94°C for 1 min, annealing at 58°C for 30 s, and extension at 72°C for 1 min; 28S: denaturation at 95°C for 5 min, followed by 35 cycles of denaturation at 95°C for 1 min, annealing at 55°C for 1 min, and extension at 72°C for 2 min). Amplification success was checked by gel electrophoresis. All three primer sets amplified in all reactions. 18S amplicons were cloned using the insTAclone PCR cloning kit (Thermo Scientific™, Waltham, MA, USA) according to the manufacturer's protocol. Plasmid DNA was extracted from overnight cultures by alkaline lysis (Sambrook, Fritsch, & Maniatis, 1989), and 40 plasmids (10 of each soil core replicate) were Sanger sequenced using vector primers (Eurofins, Konstanz, Germany). A subsequent BLAST search revealed that DNA of various organisms had been amplified, and nematodes (genus *Eucephalobus*) resembled only 2.5% of it (Table 2).

**Table 2:** Results from cloning of PCR products using soil DNA and nematode primers

Organism	%	GenBank accession numbers
Fungi	40.0	KY752080, KY752082, KY752084, KY752085, KY752088, KY752090, KY752091, KY752092, KY752096, KY752097, KY752100, KY752103, KY752104, KY752108, KY752109, KY752111
Plantae	27.5	KY752076, KY752077, KY752078, KY752079, KY752086, KY752089, KY752093, KY752095, KY752113, KY752114, KY771163
Arthropoda	10.0	KY752098, KY752101, KY752105, KY752107
Annelida	7.5	KY752081, KY752083, KY752110
Protozoa	5.0	KY752087, KY752102
Platyhelmintes	5.0	KY752099, KY752106
Bacteria	2.5	KY752094
Nematoda	2.5	KY752112
Total	100.0	

“%” is the percentage of cloned plasmids assigned to major taxonomic groups by BLAST search. The GenBank accessions refer to the sequences retrieved in this study.

## 4 The Gordian Knot of Fitting Primers

The most crucial point for the successful molecular characterization of biodiversity is the availability of suitable primers. Primers should reliably amplify the target taxa but should not bind to nontarget DNA in the sample. Here, we tested a set of nematode primers by Porazinska et al. (2009). The primers, targeting 18S rDNA, were not sufficiently specific for direct extracts or an environmental DNA approach: After cloning and sequencing of the PCR‐products, only 2.5% of the plasmids contained nematode DNA (see Box 2). Despite our small sample size, our cloning approach clearly demonstrates the lack of specificity of the available primers. This is in line with findings from Sapkota and Nicolaisen (2015) who increased the final percentage of nematode DNA among all amplicons to about 34% using a nested PCR design.

**Figure 2:**
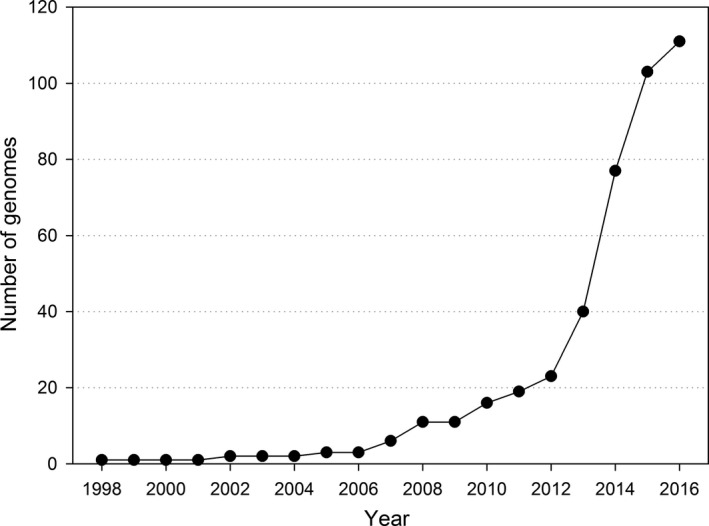
Cumulative number of whole nematode genomes available in GenBank; when the years of release and publication of the whole genome differed for a species, the earlier year was taken

There are various strategies to overcome the lack of well‐fitting nematode primers. First, other primer binding sites on the 18S gene might be more suitable. However, the huge genetic variety of nematodes (the phylum Nematoda is about 550–600 million years old, for a recent phylogeny see van Megen et al., 2009) makes the search for phylum specific, conserved regions difficult. Second, genes other than 18S might be used. A GenBank search (retrieved 05 February 2017) resulted in 983, 7,554, 16,514, and 21,736 hits for nematode COII, COI, 28S, and 18S sequences, respectively. While indeed 18S is the most often sequenced nematode gene, the currently available GenBank resource has potential to produce promising alternative alignments. A recent study comparing environmental DNA based with traditional biodiversity assessments identified the COI gene, besides 18S, as best proxy for traditional biodiversity (Drummond et al., 2015), which could be a good starting point for future work. The internal transcribed spacer (ITS) region (15,957 entries in GenBank, retrieved 05 February 2017) is another genetic marker with a long history in nematode taxonomy (Powers et al., 1997), which is probably also suitable for biodiversity assessments. Finally, 111 nematode whole genomes are currently available (GenBank, retrieved 16 March 2017), and the number of whole genome publications is currently growing exponentially (Figure 2). However, most of these genomes belong to human parasites, plant parasites of major crops, or insect parasites and are thus not relevant for soil biodiversity studies. In silico PCR may accelerate the discovery and quality control of potential new markers, as already shown in a metabarcoding approach on insects (Clarke, Soubrier, Weyrich, & Cooper, 2014). Further, whole‐genome alignments will, in the near future, create opportunities for the search of markers beyond the standard genes used today. Nevertheless, the reliability of genome data has to be critically investigated due to intragenomic polymorphism, as recently shown for marine nematodes (Dell'Anno, Carugati, Corinaldesi, Riccioni, & Danovaro, 2015).

## 5 Headlong Technological Change and Perspectives

Another rapidly changing field is the next generation sequencing (NGS) technology, which is used to analyze amplicons. Porazinska et al. (2009) used the 454 GS FLX platform (Roche Life Science, Basel, Switzerland), which has been shut down in the meantime, as announced before (Bio‐IT World Staff, 2013; GenomeWeb Staff Reporter, 2013). The various Illumina (San Diego, CA, USA) sequencers are the current workhorses in NGS, and sequencers of the third generation were launched more broadly in the last 2 years (Bleidorn, 2015). The comparatively few reads of 454 GS FLX made relative quantification of taxa quite difficult (Porazinska, Sung, Giblin‐Davis, & Thomas, 2010). Still, current sequencing technology and well‐fitting primers may sort out most of the quantification problems. Piñol, Mir, Gomez‐Polo, and Agustí (2015) found that, when sequencing COI amplicons on the Ion Torrent (Gilford, NH, USA) platform, about 75% of the variation in amplicon detection frequency arose from primer mismatches, underpinning the need of good primers. On Illumina, the use of read correction factors in metagenomic approaches becomes a routine (Thomas, Deagle, Eveson, Harsch, & Trites, 2016), and the advantages of targeted gene enrichment strategies, which forgo a PCR amplification step, were shown for freshwater macroinvertebrates (Dowle, Pochon, C Banks, Shearer, & Wood, 2016). Nevertheless, some quantification inaccuracy due to intragenomic variation of ribosomal repeats will remain (Bik et al., 2013). Several other pitfalls (e.g., temporal and spatial scale; Thomsen & Willerslev, 2015) are in need of further research. The provision of necessary and valuable information for future demands on environmental DNA, like the evaluation of soil biodiversity as a criterion for determining biodiversity in wilderness and protected areas, may represent a reward for overcoming all these hurdles (Wall, Nielsen, & Six, 2015).

## Conflict of Interest

None declared.
